# Multi-Scale Strengthened Directional Difference Algorithm Based on the Human Vision System

**DOI:** 10.3390/s222410009

**Published:** 2022-12-19

**Authors:** Yuye Zhang, Ying Zheng, Xiuhong Li

**Affiliations:** Information Science and Engineering Department, Xinjiang University, Urumqi 830017, China

**Keywords:** human visual system (HVS), infrared image, small target detection, multi-scale strengthened directional difference (MSDD)

## Abstract

The human visual system (HVS) mechanism has been successfully introduced into the field of infrared small target detection. However, most of the current detection algorithms based on the mechanism of the human visual system ignore the continuous direction information and are easily disturbed by highlight noise and object edges. In this paper, a multi-scale strengthened directional difference (MSDD) algorithm is proposed. It is mainly divided into two parts: local directional intensity measure (LDIM) and local directional fluctuation measure (LDFM). In LDIM, an improved window is used to suppress most edge clutter, highlights, and holes and enhance true targets. In LDFM, the characteristics of the target area, the background area, and the connection between the target and the background are considered, which further highlights the true target signal and suppresses the corner clutter. Then, the MSDD saliency map is obtained by fusing the LDIM map and the LDFM map. Finally, an adaptive threshold segmentation method is employed to capture true targets. The experiments show that the proposed method achieves better detection performance in complex backgrounds than several classical and widely used methods.

## 1. Introduction

Infrared imaging system have been widely used in civil fields such as diseased cell diagnosis, industrial flaw detection, and agricultural and industrial detection [1,2,3,4,5]. It is worth noting that the application value of Infrared imaging system in military fields such as military reconnaissance, early warning, guidance, and video surveillance is more obvious [4]. The infrared search and track (IRST) system is one of the core components. The IRST system refers to the system that detects the target that radiates infrared energy from the infrared image and tracks and predicts the trajectory of the target [6]. Among them, infrared small target detection and tracking is one of the core technologies of the IRST system.

However, in most practical infrared small target imaging systems, because the size of the target to be detected is very small or too far away from the detector, the target to be detected in the system output image is very small (typically no more than 80 pixels based on SPIE definition [7]) and lacks color and texture features [8,9]. Small targets in real scenes meet the following conditions: that is, the target area has observable discontinuity compared with the surrounding background area, the number of pixels in the target area is small, the contrast between the target and the background is low, the background is complex, the texture information is lacking, etc. The difficulties and challenges of many detections have attracted more and more attention from researchers at home and abroad.

At present, many infrared small target detection algorithms have been proposed at home and abroad. In general, the existing infrared small target detection algorithms can be roughly divided into single-frame algorithms and multi-frame algorithms [10]. Due to the need for early warning and the good potential of single-frame detection algorithms for real-time applications [11], this paper only focuses on single-frame-based detection algorithms. Next, we will give a brief overview of HVS-based small target detection methods and other single-frame small target detection methods.

HVS-based small target detection methods. Theoretical mechanisms such as local contrast, visual saliency map, multi-feature fusion, and multi-scale have become the new theoretical basis for infrared small target detection. In recent years, mechanisms of the human visual system have been successfully introduced into the field of infrared small target detection [12,13,14,15]. Theoretical mechanisms such as local contrast, visual saliency map, multi-feature fusion, and multi-scale have become new theoretical bases for infrared small target detection. Chen et al. proposed the local contrast measure (LCM) algorithm [12], which uses the current central region with the surrounding neighborhood for contrast measurement to obtain the contrast factor, thus enhancing the target and suppressing the background. However, this method is not suitable for detecting dark targets and has limited ability to suppress noise and background. Based on LCM, Han et al. proposed the improved LCM (ILCM) algorithm [16], which uses the subblock average as a parameter to better suppress random point noise, but true small targets may also be smoothed. Inspired by biological vision mechanisms, Wei et al. proposed the multi-scale patch-based contrast measure (MPCM) algorithm [17], which defines a local contrast measure based on patch differences for background suppression and target enhancement. Although this method is able to detect both bright and dark targets in IR images, it is not robust enough for thick clutter. Then, the novel LCM (NLCM) algorithm proposed by Tan et al. and the weighted local difference measurement (WLDM) algorithm proposed by Qin et al. [18,19] were combined, which combines the advantages of local differential contrast and local contrast to enhance the target but does not effectively suppress high brightness backgrounds. To solve this problem, Han et al. proposed the relative LCM (RLCM) algorithm [20]; however, it is more sensitive to scattering noise. Recently, Han et al. proposed the weighted strengthened local contrast measurement (WSLCM) algorithm [21], which uses the idea of matched filter and background estimation to enhance the target and suppress the background and uses a weighting function to adjust the final result, which has a better detection performance, but the time cost is high and is not suitable for real-time detecting.

Other small target detection methods. In the early days, researchers mostly worked on filter-based methods. According to the shape of the target or the background, a specific filter is constructed to achieve the purpose of enhancing the target signal and suppressing the background. For example, the top-hat filter [22], the maximum mean/maximum median filter [23], and an improved anisotropic partial difference filter [24], which can enhance the target and suppress the complex background, but the robustness is not good. Some high-order filters, such as a Laplacian of Gaussian filter [25] and bilateral filter [26], have also been designed and improved by researchers for small target detection. These algorithms are simple in design and fast in calculations. However, these methods suffer from a high false positive rate when the image signal-to-clutter ratio (SCR) is low or when the target shape is heterogeneous, thus failing to detect real targets correctly. Moreover, many scholars regard infrared small target images as the superposition of low-rank components and sparse components and propose many infrared small target detection algorithms based on robust principal component analysis (RPCA) [27]. Hu et al. [28] proposed a small target algorithm based on saliency and the principal component analysis. Cao et al. [29] proposed a small target algorithm based on a probabilistic principal component analysis (PPCA), which maps the input vector of the image to the subspace by calculating the PPCA parameters. The distance between the original vector and the reconstructed vector indicates whether the input vector is a small target or not. Gao et al. [1] proposed an infrared patch-image model (IPI), which transformed the small target detection problem into an optimization problem of recovering a low-rank sparse matrix. Generally, this type of algorithm has good robustness and a high detection rate, but it is slow in processing large-scale images and has poor real-time performance. In addition, methods based on deep learning are becoming increasingly popular research directions. Inspired by the application of generative confrontation network (GAN) in the field of unsupervised learning, Wang et al. [30] proposed a deep learning framework to balance target miss detection (MD) and false alarm (FA). Zhao et al. [31] proposed an algorithm that uses the GAN model to autonomously learn small target features, and constructs a five-layer discriminator to enhance the data fitting ability of the generator. The literature [32,33] used convolutional neural network (CNN) to propose a new infrared image enhancement method by highlighting the target and suppressing the background clutter. Generally, this type of method extracts the features of small targets in a self-learning manner to distinguish the background, trying to get rid of the tediousness of manually extracting small target features, and can improve the detection accuracy of small targets to a certain extent. However, due to the lack of large and diverse training data, such methods must generate a large dataset to simulate the properties of infrared images, including various forms of targets and backgrounds. Therefore, deep learning based methods are currently challenging.

Building on the previous work, this paper proposes a new method to enhance the directional difference. The algorithm makes full use of the anisotropy of the true target, and takes into account the features of the true target itself, the background neighborhood features, and the features between the two. Extensive data experiments show that the proposed method outperforms existing algorithms in detecting complex backgrounds. Furthermore, the method is robust to different target shapes, target sizes, and noise types.

This paper has three contributions.

1. Improved the previous scan window, the center pixel of the window does not participate in the calculation and can effectively deal with high-brightness pixel-level noise (PNHB).
2. Using the new scanning window and the anisotropy of the small target itself, a local directional intensity measure is proposed.
3. Considering the features of the true target itself, the features of the background neighborhood and the features between them, LDFM is proposed.

The article is organized as follows: In Section 2, the relative work is presented. The proposed method and its various parts are described in detail in Section 3. In Section 4, the experimental results are given, comparing the proposed method with other methods. In Section 5, the analysis and discussion of the algorithm are presented. The article ends in Section 6.

## 2. Related Work

Most of the current detection algorithms based on the mechanisms of the human visual system ignore the continuous direction information, which is a very potentially valuable information. Recently, Saed Moradi et al. [34] used a concept similar to the average absolute gray difference [35] to construct a new algorithm for directional small target detection called absolute directional mean difference (ADMD).

**Figure 1:**
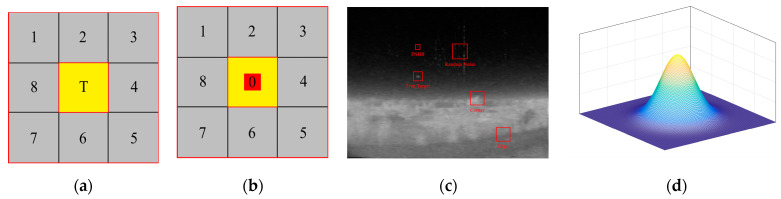
(**a**) Double-nested detection window. (**b**) Improved double-nested detection window. (**c**) Situations that the algorithm needs to handle. (**d**) Gaussian shape.

In the ADMD, first, a double nested window is defined as shown in Figure 1a, where T represents the target block, and B represents the eight background cells. The main idea of the ADMD algorithm is as follows:

(1) $$D_{k}={\left(m_{0}\left(i,j\right)-m_{k}\left(i,j\right)\right)}^{2}\times F\left(m_{0}\left(i,j\right)-m_{k}\left(i,j\right)\right)\;k=1,2\ldots 8$$

where $m_{0}$ represents average gray value of the target cell T, $\mathrm{and}\;m_{k}$ represents the average gray value of the kth background cell. F(·) is a function, as follows:

(2) $$F\left(x\right)=\{\begin{array}{c} 1\;\;\;\;\;\;\;\;\;\;\;\;\;\;\;\;\;\;x\geq 0\\ 0\;\;\;\;\;\;\;\;\;\;\;\;\;\;\;\;\;\;x<0 \end{array}$$

suppression of the negative region generated in the calculation using the F function. Since true small targets are usually brighter than their background neighbors. Therefore, ADMD is defined as follows:

(3) $$\mathrm{ADMD}\left(i,j\right)=\min\left\{D_{1}\left(i,j\right),D_{2}\left(i,j\right)\ldots D_{8}\left(i,j\right)\right\}$$

In general, the true small target area is brighter than the surrounding environment. This means that any of the selected contrast values are larger, while the non-real target area does not have this property. This definition implies the ability to enhance the target and suppress the background.

However, this method does not fully consider the anisotropy of the target point. Corner points and PNHB will have a huge impact on the algorithm, and the enhancement and background suppression of the true target are not ideal. The task goal of the algorithm proposed in this paper is to improve the ADMD algorithm, which can effectively deal with complex noise points.

## 3. Materials and Methods

**Figure 2:**
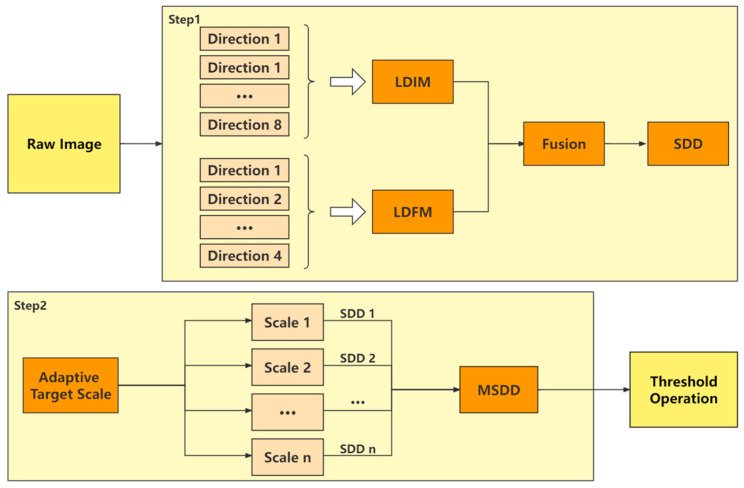
Flowchart of our MSDD small target detection method.

The flowchart of the proposed algorithm is shown in Figure 2, which mainly consists of two parts, LDIM and LDFM. First, the raw image is calculated by LDIM to obtain candidate points. Second, LDFM is used to correct wrong candidate points and enhance true small targets. Then, the LDIM map and LDFM map are fused to obtain the final saliency map (SM). Then, we extend the algorithm to multi-scale. Finally, the target is extracted by a threshold operation.

### 3.1. Local Directional Intensity Measure

In general, as shown in Figure 1d, true small targets have Gaussian-shaped features, and their gradient directions are omnidirectional—that is, the intensity decays toward the surroundings. True small targets have stronger brightness than their background neighborhood and form a higher contrast with their background neighborhood.

As shown in Figure 1b, an improved double-nested window is designed. The window is divided into 9 cells, where 0 cells are the target region, and the remaining cells are background neighborhoods. Note that the pixel position of the center point of the target area does not participate in the calculation, which can effectively avoid PNHB, as shown in Figure 1c.

Given an infrared image, candidate regions satisfying the above properties can be obtained by calculations.

(4) $$D\left(x,y\right)=\max\left\{\min\left(m_{0}-m_{i}\right),0\right\}\;i=1,2\ldots 8.$$

(5) $$m_{i}=\frac{1}{N_{i}}\overset{N_{i}}{\underset{j=1}{\sum }}G_{j}^{i}\;i=0,1\ldots 8.$$

where (x,y) is represents the center point of the target area, $m_{i}$ is the average intensity value of the ith cell, $N_{i}$ is the number of pixels of the ith cell, ${\mathrm{and}\;G}_{j}^{i}$ is the intensity value of the jth pixel in the ith cell. The min(·) and max(·) are the minimum and maximum operations, respectively. The local directional intensity measure is then obtained as follows.

(6) $$\mathrm{LDIM}\left(x,y\right)=D{\left(x,y\right)}^{2}$$

where LDIM is the square of D. This is done to enhance the true targets.

### 3.2. Local Directional Fluctuation Measure

**Figure 3:**
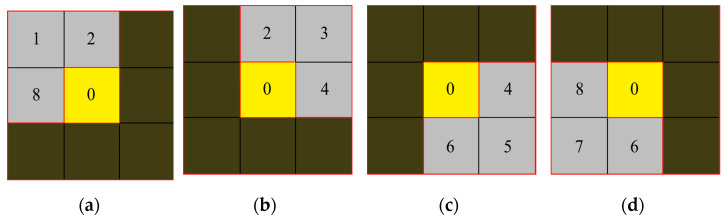
Newly divided into four directional blocks structure. (**a**) Upper left subblock. (**b**) Upper right subblock. (**c**) Lower right subblock. (**d**) Lower left subblock.

The target area, the neighborhood background, and the target-neighborhood background fluctuations should all be taken into account. Firstly, consider the fluctuation of the target-neighborhood background. In Figure 3, the three neighborhoods and the central area are taken as a piece of new area, and four blocks with directionality can be obtained after area division.

The fluctuation of each block is obtained by calculations:

(7) $$\sigma_{\mathrm{block}}\left(x,y\right)=\min\left(\sigma_{i},\sigma_{2},\sigma_{3},\sigma_{4}\right)$$

(8) $$\sigma_{i}=\sqrt{\frac{1}{N_{i}}\times \overset{N_{i}}{\underset{j=0}{\sum }}{\left(I_{j}^{i}-M^{i}\right)}^{2}}\;\;\;\;i=1,2,3,4.$$

(9) $$M^{i}=\frac{1}{N_{i}}\overset{N_{i}}{\underset{k=1}{\sum }}I_{k}^{i}$$

where $M^{i}$ is the average intensity value of the ith block. Then, the target area and the neighborhood background are calculated separately.

(10) $$\sigma_{\mathrm{center}}\left(x,y\right)=\sigma_{{\mathrm{cell}}_{0}}\left(x,y\right)=\sqrt{\frac{1}{N_{{\mathrm{cell}}_{0}}}\times \overset{N_{{\mathrm{cell}}_{0}}}{\underset{j=0}{\sum }}{\left(I_{j}-M_{{\mathrm{cell}}_{0}}\right)}^{2}}$$

(11) $$\sigma_{\mathrm{bg}}\left(x,y\right)=\mathrm{mean}\left(\sigma_{{\mathrm{cell}}_{i}}\right)\;\;\;\;i=1,2\ldots 8.$$

(12) $$\sigma_{{\mathrm{cell}}_{i}}\left(x,y\right)=\sqrt{\frac{1}{N_{{\mathrm{cell}}_{i}}}\times \overset{N_{{\mathrm{cell}}_{i}}}{\underset{j=1}{\sum }}{\left(I_{j}-M_{{\mathrm{cell}}_{i}}\right)}^{2}}$$

where mean(·) is the mean operation. Note that the calculation here is for each cell. Finally, LDFM is obtained.

(13) $$\mathrm{LDFM}\left(x,y\right)=\frac{\sigma_{\mathrm{block}}\left(x,y\right)\times \sigma_{\mathrm{center}}\left(x,y\right)}{\max\left\{\sigma_{\mathrm{bg}}{\left(x,y\right)}^{2},\zeta \right\}}$$

where ζ is a constant to prevent the denominator from being zero and is set to 0.01 in this paper.

### 3.3. Small Target Detection Using

According to the discussion in the previous section, it can be known that the LDIM map can use the characteristics of small target areas and edge areas to obtain candidate target areas in the original image and can effectively deal with edge clutter and PNHB to enhance the true target. Then, the LDFM image can be used to further suppress the corner clutter and enhance the true target. Therefore, SDD is defined as the mapping of LDIM weighted LDFM, which can greatly improve the reliability of target detection and effectively suppress the background:

(14) SDD(x,y)=LDIM(x,y)×LDFM(x,y)

In the real case, since the size of the true small target is not fixed in the IR map, it is necessary to detect the target using multi-scale operations. The proposed method can be easily extended to a suitable detection range. First, the cell size is set to different scale values. Then, the SDD at each scale is calculated, and finally, the final multi-scale SDD is achieved by the maximum operation:

(15) $$\mathrm{MSDD}\left(x,y\right)=\max\left({\mathrm{SDD}}^{\left(s\right)}\left(x,y\right)\right)\;\;\;s=1,2\ldots n.$$

where s represents the sth scale, and n represents the total number of scales.

### 3.4. Threshold Operation

In this paper, we use SDD to calculate each pixel in the original image from top to bottom and from left to right and finally get a new matrix called saliency map (SM). In SM, the true small target is the most significant, so it can be extracted by using the threshold. The threshold operation in this paper is defined as:

(16) $$\mathrm{Th}=\lambda \times {\mathrm{SM}}_{\max}+\left(1-\lambda \right)\times {\mathrm{SM}}_{\mathrm{mean}}$$

where ${\mathrm{SM}}_{\max}$ and ${\mathrm{SM}}_{\mathrm{mean}}$ are the maximum and mean values of SM, respectively. λ is an experimental constant, and its value is between 0 and 1. The experiments show that λ is the most suitable between 0.4 and 0.6. If the pixel intensity in the SM map is greater than Th, it is divided into target pixels.

## 4. Experimental Results

In this section, we test the proposed method on four infrared sequences with different background clutter, as well as open datasets, and compare the performance with six related baseline methods. The relevant performance metrics are also given in this section to verify the effectiveness and robustness of the proposed algorithm. All experiments were done with MATLAB r2016a on a computer with a 16 GB RAM and 2.50 GHz Intel i5-7300HQ processor.

### 4.1. Experimental Settings

#### 4.1.1. Related Metrics

We use background suppression factor (BSF) and signal-to-clutter ratio gain (SCRG) as metrics to evaluate the clutter suppression ability of the algorithm [35,36,37]. The definition of specific experimental metrics is as follows:

(17) $$\mathrm{BSF}=\frac{\sigma_{\mathrm{in}}}{\sigma_{\mathrm{out}}}\;\;\mathrm{SCRG}=\frac{{\mathrm{SCR}}_{\mathrm{out}}}{{\mathrm{SCR}}_{\mathrm{in}}}\;\;\mathrm{SCR}=\frac{\left|m_{t}-m_{b}\right|}{\sigma_{b}}$$

where $\sigma_{\mathrm{in}}$ and $\sigma_{\mathrm{out}}$ are the standard deviations of the original image and the significant map, respectively. ${\mathrm{SCR}}_{\mathrm{in}}$ and ${\mathrm{SCR}}_{\mathrm{out}}$ are the signal-to-clutter ratio levels of the original image and the salient map, respectively. $m_{t}$ and $m_{b}$ denote the mean values of the target area and the surrounding background area, respectively. $\sigma_{b}$ represents the standard deviation of the background neighborhood. In this paper, the target area is the area around the center of the object, and the background neighborhood is the 15 × 15 neighborhood around the center area of the object, excluding the object area. Specifically, BSF represents the degree of background clutter and noise after image processing. Specifically, BSF represents the degree of background clutter and noise after image processing. In the original image, especially the IR image with a complex background, true small targets are often submerged in it, making it difficult to be detected. Such a complex background produces a high standard deviation $\sigma_{\mathrm{in}}$. After processing, the complex background should be suppressed, tend to be flat, and, finally, get a lower standard deviation $\sigma_{\mathrm{out}}$. Therefore, the larger the ratio of $\sigma_{\mathrm{in}}$ to $\sigma_{\mathrm{out}}$, the better the background suppression effect, and the easier the detection of small targets. SCRG can indicate the degree of enhancement of the true target after processing. Usually, through the processed IR image, the intensity value of the true small target is enhanced, and the intensity value of the background neighborhood is reduced and tends to be flat. Therefore, higher values of SCRG and BSF indicate better performance in target enhancement and background suppression, respectively.

In addition, in order to better evaluate the detection accuracy of the algorithm, a threshold within a specific range is used to segment the saliency map to obtain the true positive rate (TPR) and false positive rate (FPR) and then use TPR and FPR to define the subject operating characteristics (ROC) curve [35,36,37]. The specific formulas of TPR and FPR are given as follows:

(18) $$\mathrm{TPR}=\frac{\mathrm{the}\;\mathrm{number}\;\mathrm{of}\;\mathrm{detected}\;\mathrm{true}\;\mathrm{targets}}{\mathrm{total}\;\mathrm{number}\;\mathrm{of}\;\mathrm{real}\;\mathrm{targets}}$$

(19) $$\mathrm{FPR}=\frac{\mathrm{the}\;\mathrm{number}\;\mathrm{of}\;\mathrm{detected}\;\mathrm{false}\;\mathrm{targets}}{\mathrm{total}\;\mathrm{number}\;\mathrm{of}\;\mathrm{pixels}\;\mathrm{in}\;\mathrm{the}\;\mathrm{whole}\;\mathrm{image}}$$

In the ROC curve, the more the curve is shifted to the upper left corner, the better the detection performance is, and the more it is shifted to the lower right corner, the weaker the detection performance is.

#### 4.1.2. Test Datasets and Baseline Method

The experiment uses five datasets to evaluate the performance of the algorithm, including four consecutive sequences and a set of single-frame infrared images. Among them, sequence datasets 1–4 are shown in [37,38,39]. Sequence 1 is a sequence of pictures with a single small target, and there are more PNHBs in the background. The infrared image of sequence 2 is heavily polluted by noise. In sequence 3, fliers were submerged in a complex background. In sequence 4, objects fly through the sky and buildings under overexposed conditions. For the last set of single-frame infrared images, we use the open dataset SIRST initiated by Dai et al. [40]. This dataset is the first to explicitly build an open single-frame dataset by selecting only one representative image from the sequence [40]. It is worth noting that SIRST contains small targets of different sizes, different types, different brightness, and different backgrounds. Small target types include aerial objects, ships, vehicles, etc. The background of the small target includes clouds, ground, rivers, buildings, etc. In general, the five datasets selected can test the detection ability of the algorithm and the robustness of the algorithm. Other details of the dataset are shown in Table 1.

To better evaluate the performance of the proposed method, some classical, as well as newer infrared small target detection algorithms, are selected for comparison in this paper, including LCM [12], MPCM [17], RLCM [20], ADMD [34], TLLCM [36], and VAR-DIFF [37].

### 4.2. Comparison to Baseline Methods

**Figure 4:**
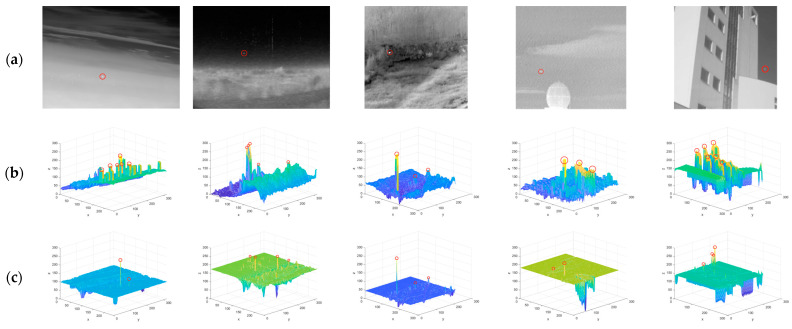

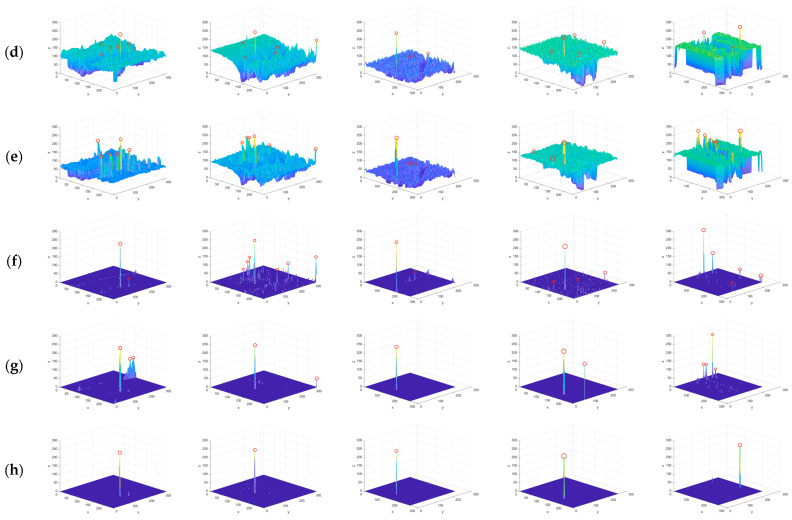
Processing results of different algorithms. (**a**) Original image, (**b**) LCM, (**c**) MPCM, (**d**) TLLCM, (**e**) RLCM, (**f**) ADMD, (**g**) VAR-DIFF, and (**h**) proposed method. Note that the gray values of all images are normalized to the [0–255] interval.

We selected one representative image in each dataset. Using the baseline method and the method proposed in this paper to compare, and the final calculation result is shown in Figure 4. In each dataset, the size of small targets is not fixed, and the background is complex, accompanied by varying degrees of noise. The first image contains more PNHB and sharp edges, which cause some baseline methods to fail to detect correctly, and more noise remains. The second image contains more target-like points, and the true small targets have a small contrast with their background neighborhoods. The true small targets are submerged in the background, and most baseline methods cannot handle the target-like points. The background of the third image is complex, but the true small target has a large contrast with its background neighborhood, so most of the baseline methods can effectively detect the true small target. The fourth picture is brighter overall, and there are buildings, and some baseline methods fail. The fifth picture contains complex buildings with many corners, and the true small target has a small contrast with its background neighborhood. Except for the algorithm proposed in this paper, all baseline methods cannot be detected normally. In a word, the algorithms proposed in this paper can effectively capture true small targets, and the effect is better than the baseline method.

As shown in Table 2 and Table 3, the proposed algorithm has good performance. Among the four sequences and SIRST, our method is the best in terms of the SCRG compared to the baseline methods. In terms of the BFS, the proposed algorithm performs well in sequence 1, sequence 2, and SIRST and is lower than VAR-DIFF in sequence 3 and sequence 4. This shows that the proposed algorithm has better target enhancement ability and background suppression ability than the other algorithms.

**Figure 5:**
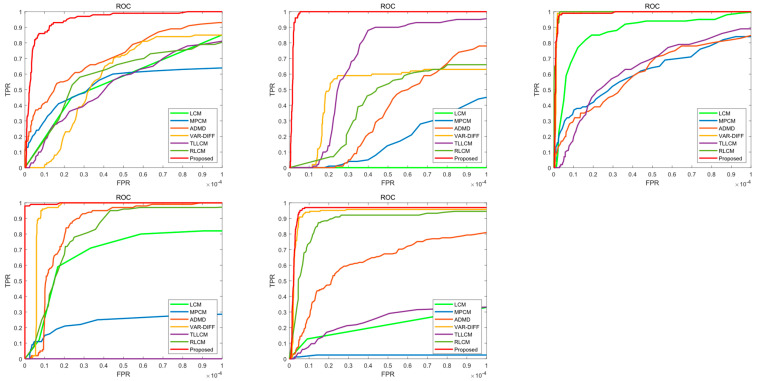
ROC curves of the four sequences and SIRST.

In addition, in order to show that the algorithm proposed in this paper has a good detection ability, we use the receiver operating characteristic (ROC) curve to conduct experiments. In Figure 5, the ROC curves for the four sequences and SIRST using the baseline method and our method are shown. It can be seen that the algorithm proposed in this paper performs well in sequence 1, sequence 2, sequence 4, and SIRST, and the rest of the baseline methods will be affected by different degrees of background clutter, resulting in algorithm instability. In sequence 3, the proposed algorithm and the VAR-DIFF and RLCM baseline methods perform well.

## 5. Discussion

Different degrees of detection interference that may be encountered need to be discussed. Next, we discuss the different cases when the window is on the true target, pure background, background edge, corner edge, and PNHB.

1. If (x,y) is true target center, since the true small targets usually has a large positive contrast to its neighborhood, its D will be large, so its LDIM will be large. Meanwhile, its $\sigma_{\mathrm{block}}$ and $\sigma_{\mathrm{center}}$ will be large, and $\sigma_{\mathrm{bg}}$ will be small, so its LDFM will be large. Therefore, the MSDD will be large.
2. If (x,y) is pure background, since the pixel intensity values of such area are not very different, its D will be so small as to be close to 0, so its LDIM will be small. Meanwhile, its $\sigma_{\mathrm{block}}\approx \sigma_{\mathrm{center}}\approx \sigma_{\mathrm{bg}}$, so its LDFM is approximately equal to 1. Therefore, the MSDD will be small.
3. If (x,y) is background edge, since such regions are usually directional locally, its D will be small, so its LDIM will be small. Meanwhile, its $\sigma_{\mathrm{block}}\approx \sigma_{\mathrm{center}}\approx \sigma_{\mathrm{bg}}$, so its LDFM is approximately equal to 1. Therefore, the MSDD will be small.
4. If (x,y) is a corner edge, such areas often appear at the edges of clouds or buildings. Particularly, these regions tend to have large positive contrasts with certain neighborhoods. However, in LDIM, the computation is directional, so the LDIM will be small. Meanwhile, $\sigma_{\mathrm{block}}$ is also a directional calculation. Therefore, its $\sigma_{\mathrm{block}}$ will be small. Overall, the final ${\mathrm{MSDD}}_{\mathrm{corner}}$ will be smaller than the ${\mathrm{MSDD}}_{\mathrm{real}\;\mathrm{target}}$.
5. If (x,y) is a PNHB, although it has high brightness characteristics, its size tends to be a single pixel. Since the center point of the newly constructed target window does not participate in the calculations, the proposed algorithm is able to handle this special case. The specific calculation results in this case requires a specific area, and we can refer to the above situation.

From the above discussion, it can be seen that the algorithm proposed in this paper is robust and can effectively deal with different interference situations.

## 6. Conclusions

This paper proposes a detection algorithm for infrared small targets using local direction differences for target enhancement and background suppression, considering the target features, background neighborhood features, and the relationship between the two and then performing multi-scale fusion calculations, and, finally, using the threshold operation to get the true target. The experimental results show that the algorithm has good detection performance for small true infrared targets of different sizes, types, and brightness, and has achieved satisfactory results in the detection rate, signal-to-noise ratio gain, and background suppression.

## Figures and Tables

**Table 1:** Information of the test datasets.

Datasets	Frames	Resolution	Target Size	Target Details	Background Details
Seq-1 [39]	100	320 × 240	5 × 5 to 7 × 7	Keeping little motion	Multiple PNHB
				Small in size	Heavy noise
Seq-2 [38,39]	100	320 × 240	5 × 5 to 7 × 7	Keeping motion	Complex clouds
				Low SCR value	Heavy noise
Seq-3 [37]	100	256 × 256	5 × 5 to 7 × 7	Keeping motion	Multiple complex objects
				Irregular shape	Heavy noise
Seq-4 [38]	100	256 × 239	3 × 3 to 7 × 7	Keeping motion	Multiple buildings
				Low SCR value	Heavy noise
SIRST [40]	427	Variety	3 × 3 to 11 × 11	Variety	Variety

**Table 2:** SCRG of different algorithms.

Datasets	LCM	MPCM	RLCM	TLLCM	VAR-DIFF	ADMD	Proposed
Seq-1	2.9678	7.1558	4.9549	3.4675	143.2399	45.8036	**220.5620**
Seq-2	2.2637	4.0981	4.8788	7.9981	40.2805	90.1688	**174.6367**
Seq-3	2.8371	4.4802	10.0950	5.7227	65.7572	65.5795	**208.1402**
Seq-4	1.2835	4.4898	2.1285	2.9342	35.1933	19.5947	**97.7643**
SIRST	2.0452	3.7768	3.8955	3.1540	47.3826	42.0477	**100.4070**

**Table 3:** BFS of different algorithms.

Datasets	LCM	MPCM	RLCM	TLLCM	VAR-DIFF	ADMD	Proposed
Seq-1	1.2985	6.5079	3.1104	2.1061	32.7192	93.1016	**653.6033**
Seq-2	1.3230	7.3366	3.5213	1.9475	54.6992	25.6519	**222.1676**
Seq-3	2.3014	10.8582	3.8682	3.5872	**179.8385**	34.4299	132.4181
Seq-4	1.2641	4.3856	3.4904	2.7104	**695.0363**	44.2576	425.8237
SIRST	1.4672	8.2238	3.9480	2.6238	1.5051 × 10^3^	189.7067	**1.6933 × 10^3^**

## Data Availability

Not applicable.
